# Modelling the potential of genetic control of malaria mosquitoes at national scale

**DOI:** 10.1186/s12915-019-0645-5

**Published:** 2019-03-29

**Authors:** Ace R. North, Austin Burt, H. Charles J. Godfray

**Affiliations:** 1 Department of Zoology, University of Oxford, Oxford, UK; 2 Imperial College, London, UK; 3 Oxford Martin School, University of Oxford, Oxford, UK

**Keywords:** Malaria, Driving-Y, Gene-drive, Mosquito

## Abstract

**Background:**

The persistence of malaria in large parts of sub-Saharan Africa has motivated the development of novel tools to complement existing control programmes, including gene-drive technologies to modify mosquito vector populations. Here, we use a stochastic simulation model to explore the potential of using a driving-Y chromosome to suppress vector populations in a 10^6^ km^2^ area of West Africa including all of Burkina Faso.

**Results:**

The consequence of driving-Y introductions is predicted to vary across the landscape, causing elimination of the target species in some regions and suppression in others. We explore how this variation is determined by environmental conditions, mosquito behaviour, and the properties of the gene-drive. Seasonality is particularly important, and we find population elimination is more likely in regions with mild dry seasons whereas suppression is more likely in regions with strong seasonality.

**Conclusions:**

Despite the spatial heterogeneity, we suggest that repeated introductions of modified mosquitoes over a few years into a small fraction of human settlements may be sufficient to substantially reduce the overall number of mosquitoes across the entire geographic area.

**Electronic supplementary material:**

The online version of this article (10.1186/s12915-019-0645-5) contains supplementary material, which is available to authorized users.

## Background

A major scaling-up of measures to reduce the global burden of malaria between 2000 and 2014 resulted in substantial reductions in disease prevalence [1]. Since 2014, however, this progress has stalled and even reversed in some regions [2]. There are two main factors behind this setback. First, there are major and persistent geographic gaps in access to control measures [2]. Currently, the most important control measures are insecticide-treated bed nets (ITNs) that reduce the risk of infection and artemisinin-based combination therapy (ACT) that is used to treat clinical cases of *Plasmodium falciparum* malaria [1, 2]. In 2016, ITN coverage was just 54% among people at risk of malaria in Africa [2], while coverage of ACT drugs across different sub-Sahara African countries in 2014 ranged from 8 to 72% [3]. Second, neither insecticides nor anti-malaria drugs are fully effective at interrupting malaria transmission. ITNs protect against vector mosquitoes biting indoors when people are asleep yet outdoor biting is frequent in some populations [4]. Treatment of clinical malaria with ACT is only given to symptomatic patients, yet it is thought that much malaria transmission results from asymptomatic infections [5]. It is of particular concern that biological resistance has evolved in both mosquito vectors and malaria parasites [2]. While parasite resistance to ACT is currently confined to the Greater Mekong Subregion of South-East Asia [6, 7], mosquito resistance to pyrethroids (the most common group of insecticides used in bed nets) is now widespread across sub-Saharan Africa [8, 9]. There is a compelling need to develop new anti-malaria tools to complement current interventions.

One promising novel approach is to use gene-drive technologies to spread desirable traits into vector populations [10]. Gene drives are genetic constructs that positively bias their own inheritance and thus spread rapidly through populations, even if they incur a fitness cost. The general idea of using gene drive to control malaria vector populations dates back to the 1960s [11] but received new impetus with the discovery of driving-endonuclease genes (DEGs) and molecular advances that allowed their introduction into insects. Most recently, the use of CRISPR/cas9 technology to create novel DEGs has further spurred interest [12]. There are two main strategies to use DEGs to reduce disease transmission [10]. First, in population suppression DEGs are used to spread a genetic element that imposes a fitness cost on the vector, thereby causing population decline or even collapse. Second, in population replacement, a DEG is used to replace a current genotype with one less able to transmit disease. In this paper, we consider one of the most promising approaches to population suppression, the use of a ‘driving-Y chromosome’ [13]. An endonuclease is placed on the Y chromosome and engineered so that it cuts the X chromosome at one or more locations. The endonuclease’s expression is governed by a promoter active during meiosis which means that a high proportion of X gametes are destroyed and the individual’s sperm are largely Y gametes. The driving-Y spreads because it is represented in more than half its bearer’s offspring (and so is competitively superior to a normal Y chromosome).

Mathematical models show that a driving-Y chromosome can rapidly increase in frequency following a small release into a population, leading to a male-biased sex ratio and population suppression or even elimination. Spatially unstructured models demonstrate that the higher the X-chromosome cleavage rate, the greater the sex ratio bias [14, 15]. Population elimination will occur if the sex ratio bias is so great that a female mating a male carrying a driving-Y chromosome on average produces less than one daughter that survives to reproduce. This suggests that control will be harder for species with high fecundity. These general conclusions are robust to the addition of simple forms of spatial heterogeneity [16, 17] and seasonality [17, 18] though these factors may affect the detailed outcomes. In contrast with some current vector control tools such as ITNs, the most challenging environments for driving-Y control appear to be those with sparse mosquito habitat and strong seasonality, such that mosquitoes tend to aggregate in small local populations with little mixing [16, 17]. Releases of driving-Y mosquitoes at the start of the rainy season may help offset the difficulty of spread in such environments [17, 18].

Current spatially explicit models deal with scales of less than 1000 km^2^ while vector control authorities need to consider country-scale deployment over regions with considerable spatial and temporal variation. Here, we consider a region of one million square kilometres that includes all of Burkina Faso and parts of the surrounding West African countries. This area has strong seasonality in rainfall, a pronounced north-south cline in total rainfall, and large variations in human population density. We investigate population suppression by incorporating driving-Y chromosomes into a model that we have previously used to explore how mosquito populations persist in regions with long and severe dry seasons [19]. The model was designed to represent *Anopheles gambiae* and its morphologically indistinguishable sister species *An. coluzzii*, which are probably the most efficient malaria vectors in the world [20]. Both species are strongly anthropophilic [20–23], and for this reason, the model assumes that all mosquito populations in the study area are associated with human settlements. It is also assumed that populations are regulated by competition for resources among larvae and that the strength of larval competition is influenced by rainfall and the distribution of groundwater along riverbanks and lake edges. We asked whether mosquito populations rebound quickly after the dry season because of (i) dispersal from populations associated with the nearest permanent water body [24–27], (ii) low levels of breeding during the dry season in cryptic small water bodies [28], (iii) aestivation by females [29–36], or (iv) long-distance migration using high altitude wind currents [29]. We found that (i) alone could not explain observed patterns of mosquito abundance in the area while the other three could with (iv) marginally the best at reproducing the observations [19].

To incorporate driving-Y chromosomes into our model [19], we suppose that any given male mosquito carries either a driving or normal Y-chromosome. The Y-chromosome is invariably inherited in male offspring, and we assume it determines the offspring sex ratio. We investigate how vector population suppression is influenced by the driving-Y chromosome release strategy, the rate of mosquito movement, and the X-chromosome cleavage rate. In addition, we ask how driving-Y chromosome spread is affected by different assumptions about dry season mosquito persistence.

## Results

### Is population suppression by a driving-Y chromosome logistically feasible?

To begin, consider a programme of releasing transgenic male mosquitoes at a number of sites (settlements) over several years. We suppose that release sites are chosen at random (from a total of 42,360 possible settlements) and that, in every site, the date of release during the year is also random. If releases of just 10 transgenic male mosquitoes take place at 1% of all the sites in our study area each year, our model predicts on average 93.6% population suppression after 4 years (95% of simulations were in the range 91.4–95.5%). By contrast, a Sterile Insect Technique (SIT) programme where 50,000 sterile male mosquitoes are released at every site once per year results in only 0.40% suppression (0%-0.94%). SIT releases at this frequency, despite the very large numbers of insects liberated, are ineffective because of the short generation time of anopheline mosquitoes. To simulate both driving-Y and SIT campaigns, we assumed that the released males have equal mating ability to wildtype males, and to simulate SIT, we assumed that females mated to released males are completely infertile.

**Fig. 1.**
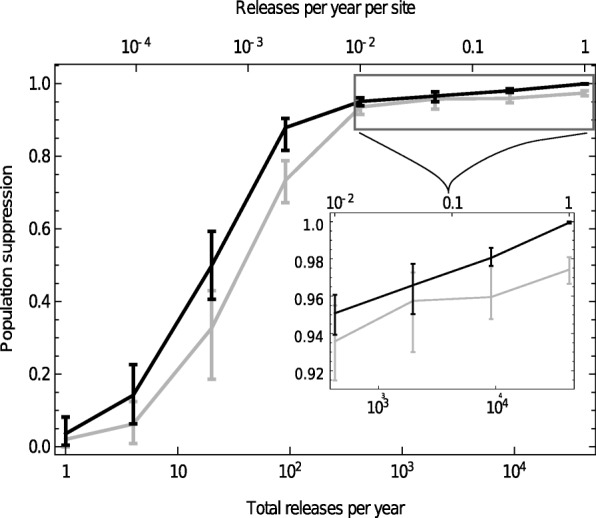
Population suppression after 4 years of driving-Y releases. Suppression depends on how many releases occur each year, and the number of mosquitoes in each release. Suppression is computed as the fractional reduction in adult female population size across the study area, and the bars indicate the ranges of 95% of simulation results for each parameter set (from > 50 simulations; bounds are 2.5% and 97.5% quantiles)

Levels of suppression are predicted to increase as releases are made at a greater percentage of sites though beyond 1% the marginal benefits decrease. The model suggests that making releases in around 1 in a 100 settlements is a good compromise between release effort and impact (Fig. 1). At this release coverage, the outcome is relatively insensitive to the number of mosquitoes released per site (releases of 10 or 5000 male mosquitoes have similar impact). As long as sufficient mosquitoes are released to overcome the risk of stochastic extinction of the driving-Y chromosome (which could in the field be greater than in the model), the size of each release does not seem to be too critical. A real control programme would obviously not release in randomly chosen settlements and we found slightly higher suppression (95.9%; 93.7–97.5%) in a stratified programme where 424 release sites were selected on an approximate lattice (Additional file 1: Figure S1). In the rest of the paper, we assume releases of 10 transgenic male mosquitoes in 1% of sites, chosen randomly.

The mosquito population suppression achieved during the programme persists after releases stop, increasing slightly from 93.6% to 94.1% (90.7–97.7%) after 8 years. The driving-Y chromosome is maintained in the landscape (see below), and further releases, at least at random locations, do not improve outcomes (Additional file 2: Figure S2).

### Is population suppression geographically uniform?

**Fig. 2.**
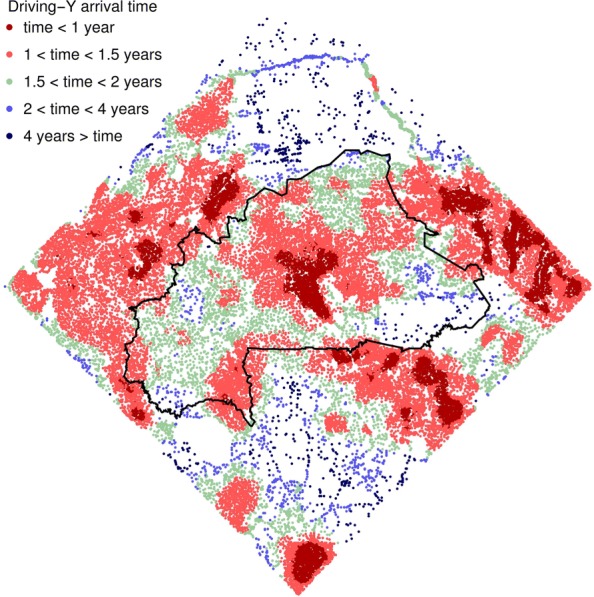
The rate of driving-Y spread through the study areas. The black line demarcates Burkina Faso, and the colours show the time it takes for driving-Y chromosomes to first arrive at each site after releases begin (average over 50 simulation runs and after excluding release sites)

In the absence of driving-Y chromosomes, we expect considerable variation in the dynamics of local mosquito populations, due to differences in the distribution of permanent groundwater, rainfall, and the connectedness of local populations. Further variation may stem from the variable application of existing vector control measures, in particular bed nets. This heterogeneity influences the spread of the driving-Y chromosome with most rapid advances occurring in densely populated parts of the landscape where dispersal between neighbouring settlements is frequent (Fig. 2). With our default assumptions, all but the most isolated settlements receive driving-Y chromosomes within 4 years of releases (98.7% of settlements; 98.6–99.1%), though we note that this result is sensitive to the parameterisation of dispersal, something we return to below.

**Fig. 3.**
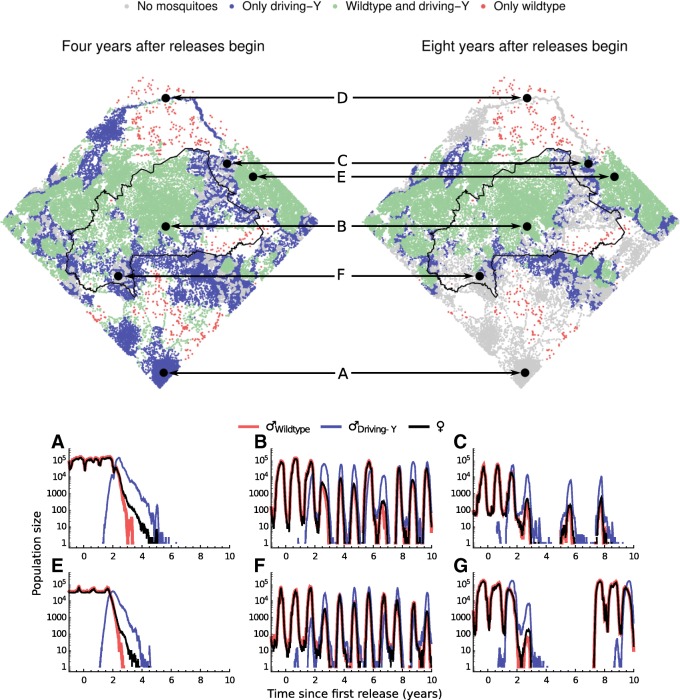
The impact of a driving-Y chromosome across the study area. The upper plots show the most common type of population (from > 50 simulation runs) at each site 4 years and 8 years after releases begin. The lower plots show the dynamics of a single typical simulation at the featured sites, in terms of the numbers of adult males (wildtype or driving-Y) and adult females

Regional heterogeneity also influences the fate of populations after driving-Y chromosomes arrive (Fig. 3). First, consider the densely populated southernmost part of the study area (A in Fig. 3, part of Ghana), and the river Niger in the northernmost part of the study area (D in Fig. 3, part of Mali). These are typical of sites where driving-Y chromosomes steadily increase in frequency after their arrival, becoming fixed within 2–4 years and leading to population elimination within another 2–3 years. These and other sites where fixation is followed by elimination by about year 8 are characterised by year-round mosquito breeding, either because of relatively low rainfall seasonality (as in site A) or the presence of permanent water bodies (as in site D).

A second type of dynamics is predicted to occur in central Burkina Faso (B in Fig. 3) and Western Niger (E in Fig. 3), where dry-season conditions are severe leading to very low mosquito population numbers at this time of year. Stochastic elimination is frequent in the absence of the driving-Y chromosome and occurs more often in those populations where the chromosome is present. At the start of the wet season, purely wildtype populations are at an advantage because they can grow faster and are more likely to colonise sites with no mosquitoes, while within a mixed population the driving-Y chromosome will replace the normal Y. The result of the between-population advantage of the normal Y chromosome and the within-population advantage of the driving-Y chromosome, played out on a spatial landscape, is a balance where both genetic variants persist in a dynamic metapopulation. Sites of this type are thus characterised by high rates of elimination (especially of populations containing driving-Y chromosomes) and high connectivity (many nearby settlements), features that allow the normal Y to survive in the ensemble even when it is outcompeted locally. We note that although mosquitoes are not eliminated at these sites, their population densities are typically significantly below their pre-intervention state.

A third type of dynamics is seen in regions with moderate variation in the availability of mosquito breeding sites, for example in Niger close to the Burkina Faso border (C in Fig. 3) and in Southern Burkina Faso (F in Fig. 3). Here the severity of the dry season is mitigated by proximity to perennial rivers. Typically, the driving-Y chromosome spreads to fixation and causes local elimination, as in the first type of dynamics. However, elements of the second type of dynamics also occur and the occasional wildtype population survives providing mosquitoes that can disperse and recolonise empty patches (the source of colonists may also be adjoining highly seasonal areas with the second type of dynamics). The result is again a dynamic balance but one in which periods of competition are punctuated by times when no mosquitoes are present. Average populations over time are significantly below those in the absence of intervention.

### Is local dispersal rate important to driving-Y chromosome impact?

**Fig. 4.**
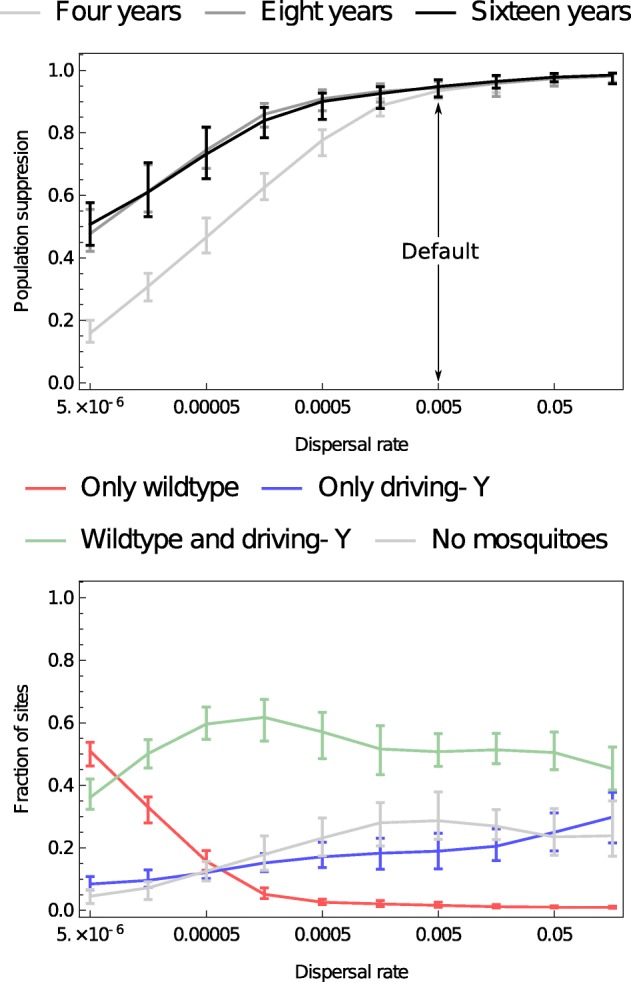
The role of dispersal. The rate at which mosquitoes disperse among neighbouring settlements influences both short- and long-term population suppression (top) and the numbers of each type of population (lower; 8 years after releases begin)

A driving-Y chromosome spreads from its release locations as mosquitoes move among neighbouring settlements, and a higher rate of dispersal accelerates this process. We found that higher rates of dispersal increased the extent of suppression until a threshold, beyond which there was little further increase (Fig. 4; see Additional file 3: Figure S3 for how dispersal influences the spatial distribution of population types). The time taken to reach the equilibrium level of suppression was greater for low levels of dispersal though by 8 years convergence had occurred in all simulations.

Increasing dispersal has two effects on Y-chromosome dynamics. First, it allows the driving-Y chromosome to colonise new patches before its current patch goes extinct. This acts in favour of driving-Y chromosome spread and population suppression. Second, it allows more productive (in terms of dispersing individuals) normal-Y populations to colonise empty patches so promoting the coexistence of the two chromosomal types in circumstances where a dynamic metapopulation occurs. This will tend to act against population suppression. Of course, as dispersal becomes very high, the predictions of non-spatial models (e.g. [14]) are obtained as a limiting case.

Unfortunately, there have been only a few attempts to estimate the rate of mosquito dispersal between settlements in West Africa. We are aware of three mark-release-recapture experiments of *An. gambiae* or *An. coluzzii* that have observed movements between villages in West Africa [37–39]. These data suggest such movements are rather frequent, translating to a dispersal rate in the range of *d*=0.005−0.034 (probability of an adult mosquito dispersing to a neighbouring village less than 12 km away on a given day) [19]. We have set our default dispersal rate at the lower end of this range (*d*=0.005).

### How does dry season mosquito ecology affect driving-Y chromosome spread?

Many sites in the study area (∼ 60*%*) receive virtually no rainfall during the dry season and are some distance from any permanent water body. This raises the question of how populations persist in the likely absence of larval breeding sites. Our previous study [19] concluded that realistic levels of dispersal from the nearest site with permanent breeding habitat could not explain the near-ubiquity of mosquito vector populations during the subsequent wet season, and examined three alternatives. Here, we explore the effects of these different assumptions about mosquito ecology on driving-Y chromosome suppression.

**Fig. 5.**
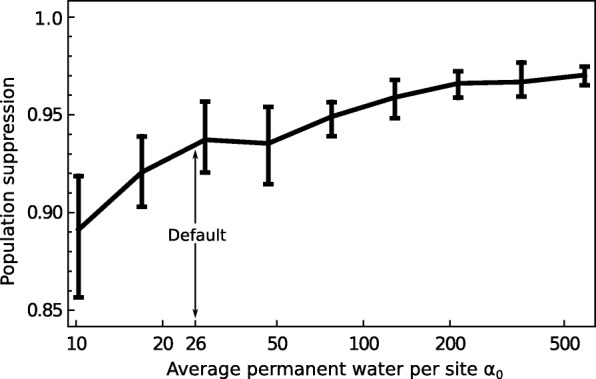
The role of unknown small permanent water bodies. By default, we assume there is a low density of small water bodies that maintain populations through the dry season (*E*[*α*_0_]=26, meaning the probability a juvenile survives larval competition is > 0.5 over the course of development if there are < 26 larvae in a population)

First, there may be small water bodies present during the dry season that are too tiny to be listed in the permanent water database that we used to parameterise the model. In our default simulations, we assumed enough were present so that in the absence of interventions the predicted wet season mosquito distribution matched field observations. The extent of population suppression caused by a driving-Y increases with the abundance of these small breeding sites (Fig. 5). The reason for this is that their presence reduces the rate of stochastic elimination of small populations during the dry season and so tilts the balance towards the driving-Y chromosome causing area-wide elimination rather than coexistence in a dynamic metapopulation.

Second, adult females might aestivate during the dry season in sufficient numbers to re-establish populations in the following rainy season. To investigate this possibility, we assume that there are no small water bodies in the dry season and set the probability of a female surviving aestivation at 0.1, the value at which predicted wet season mosquito distributions match field observations. In comparison with the default assumptions, the extent of population suppression after 4 years of releases is now less (89.5% suppression; 95% of simulations in the range 85.2–92.4%) though this difference diminishes with time and disappears after 8 years (95.7%; 92.7–97.6%, versus 94.2%; 91.3–97.8%). The effects of aestivation are qualitatively similar to those of the presence small water bodies except that the inactivity of mosquitoes over the dry season lessens the number of generations per year which slows the driving-Y chromosome’s speed of spread.

The final possible factor we consider is the long-distance migration of adult female mosquitoes using high-altitude winds. Again, we assume there are no small permanent water bodies in addition to those derived from the watercourse data and a probability of surviving migration (0.01) that results in all sites being occupied each rainy season. In contrast to aestivation, the initial population suppression is greater if migration is assumed (96.9% (91.5–99.1%) after 4 years), yet the 8-year suppression is again similar (96.1% (91.7–99.5%)). Migration accelerates driving-Y spread into remote locations giving rise to a more rapid impact.

**Fig. 6.**
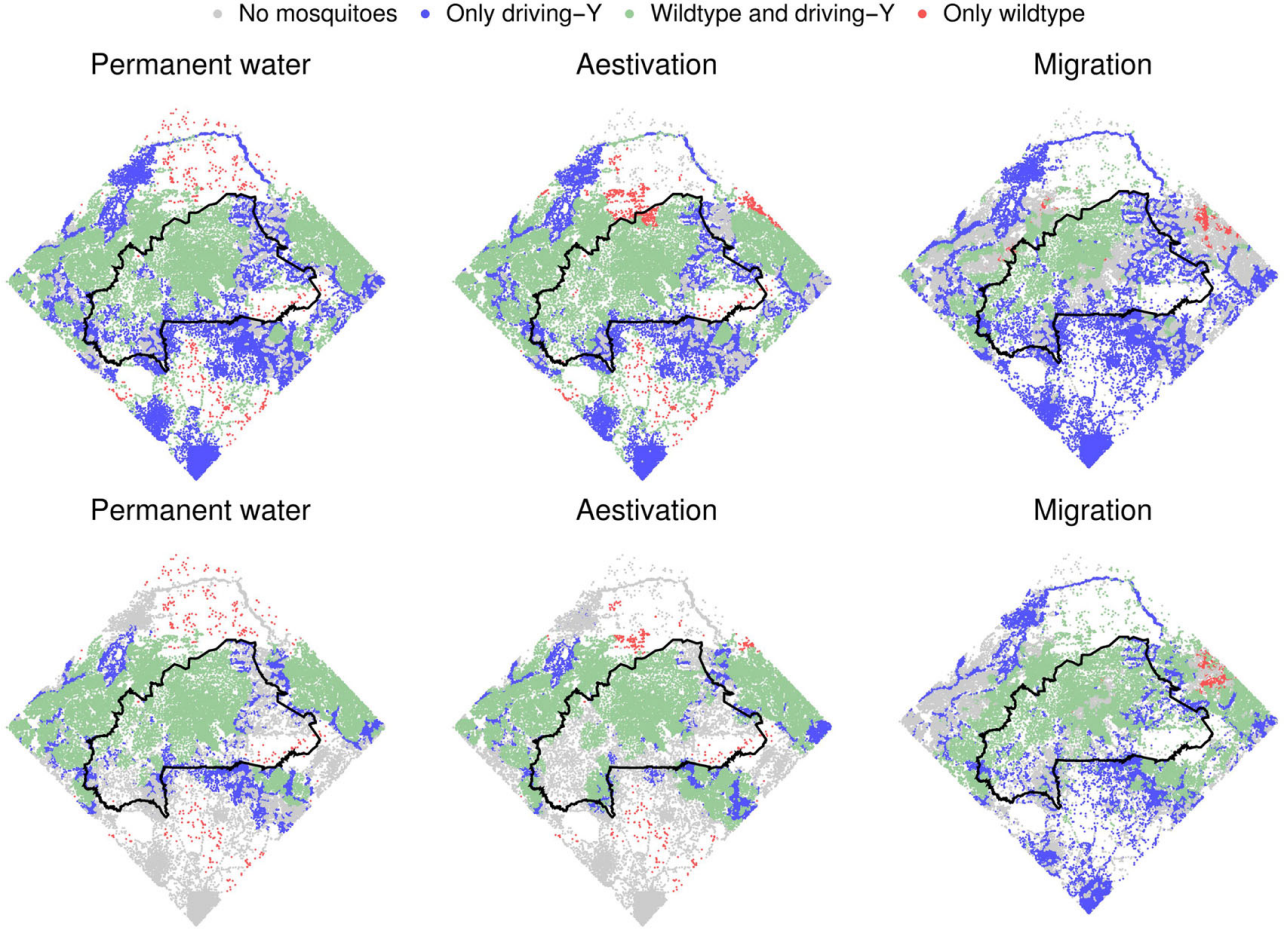
The role of mosquito dry season ecology. Upper panels show mosquito distributions 4 years after releases began and lower panels after eight years

Though the long-term impact on population size is similar, the distribution of Y-chromosomes in the landscape differs markedly depending on dry season ecology (Fig. 6). In particular, migration causes Y-chromosome mixing along the SW-NE migration routes, which has the dual effect of accelerating driving-Y movements into wildtype populations and promoting the recolonisation of regions that are otherwise predicted to be extinct. In result, empty sites and exclusive wildtype populations are both more common after 4 years if migration is occurring (Additional file 4: Figure S4).

### Driving-Y cleavage and the population growth rate

**Fig. 7.**
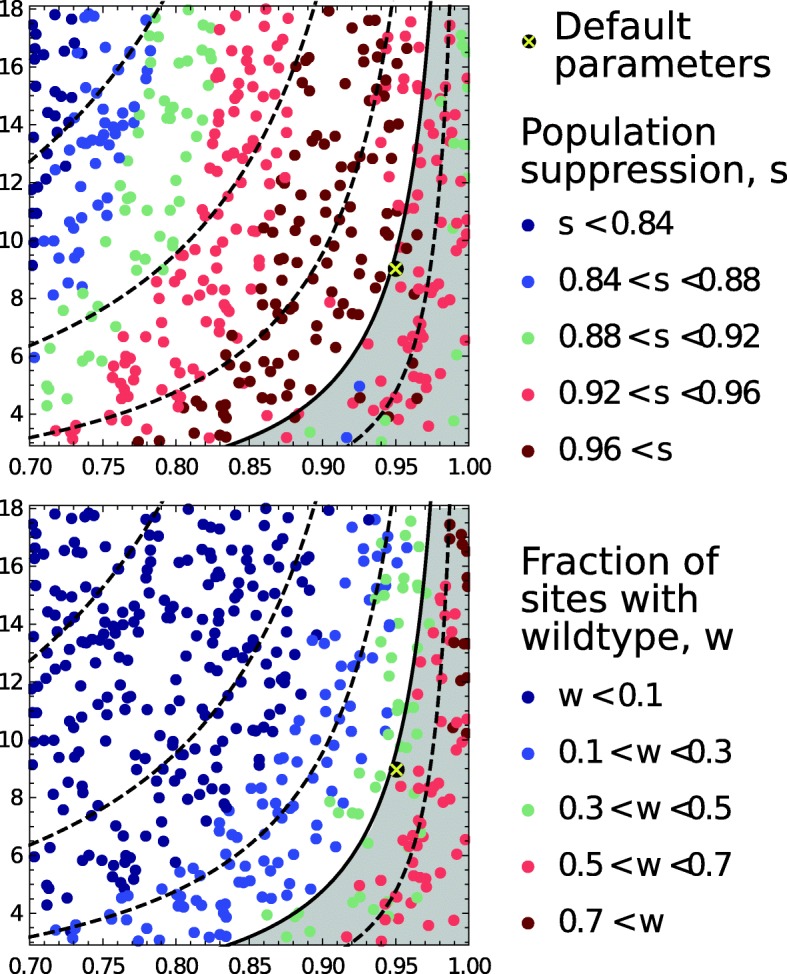
The role of driving-Y cleavage rate and the background population growth rate. These parameters influence population suppression (top; 8 years after releases begin) and wildtype presence (bottom; the fraction of sites where there are normal Y-chromosomes). The contours chart the growth rate of a population fixed with driving-Y chromosomes, at values 8, 4, 2, 1 (solid line) and 0.5 from left to right, computed from a simple model of a well-mixed population (described in [15])

Simple models assuming a well-mixed population predict that the extent of population suppression depends on the intrinsic population growth rate and the probability of X-chromosome cleavage [14, 15]. We investigated the effect of these two parameters in our more complex spatial model (Fig. 7). The solid line in the figure is the elimination threshold predicted by the simple model. As is also found in the simple model, we observed population suppression increasing as the line is approached from top to bottom (population growth rate drops) or from left to right (cleavage frequency increases; Fig. 7a). However, beyond the threshold (the grey area), the trend reverses with suppression now decreasing as further changes to these two parameters are made. A different pattern is seen by the distribution of the normal Y chromosome; decreasing the population growth rate and increasing the cleavage rate both result in a greater fraction of sites harbouring wildtype mosquitoes (Fig. 7b).

Outside the grey area in the figures, local population extinctions occur relatively rarely and the driving-Y chromosome spreads to fixation across the entire landscape causing a degree of suppression that can be estimated using the simple, non-spatial models. But as suppression increases and the critical threshold is approached, local extinctions become more likely, especially in regions with severe dry seasons. This allows the type of metapopulation dynamics described earlier with both chromosome variants present to occur. As extinctions become more common, the normal Y chromosome increasingly benefits from the higher productivity of wildtype populations and the reduced risk of being invaded by the driving-Y chromosome, leading to reduced suppression and a higher fraction of wildtype populations. Our simulations suggest that the optimal levels of population suppression occur when cleavage rates are just below the critical threshold.

Note that while we have assumed that carrying capacity is set by the availability of breeding habitats at a site, itself determined by rainfall and the presence of permanent water bodies, we assumed here that the intrinsic population growth rate is constant in space and time. In the supplementary material, we report that allowing egg-laying rate and carrying capacity to co-vary with breeding habitat availability does not affect our conclusions (Additional file 5: Figure S5).

## Discussion

Providing regulatory agencies approve its deployment, gene-drive technology holds great promise for malaria vector control. However, the potential spread and impact of DEGs in realistic landscapes has received insufficient study. Here, we investigated the use of a suppression gene drive across an area of West Africa that is large enough to exhibit the wide variation in environmental conditions found in this region. We find the impact is likely to vary across the region, yet substantial and widespread suppression of vector populations can be achieved from a logistically feasible programme of releases.

Our model identified regions where neither the driving-Y nor the normal-Y chromosome is able to displace the other in all local sites simultaneously due to the complex dynamics of local extinction and recolonisation. Similar dynamics were found in spatial models of driving-Y chromosome releases in simulations of a ∼ 1000- km^2^ area of Nigeria [17], and in ∼ 250- km^2^ artificial landscapes [16]. In some regions we simulated, the driving-Y chromosome completely replaces the normal chromosome and the dynamics are well approximated by models without spatial structure [14, 15]. We also found regions where the dynamics were somewhat intermediate, with population sites typically unoccupied for several years with sporadic episodes of recolonisation and extinction. Our model shows that different types of dynamic behaviour can occur within regions of the size we study and that consideration of how these subregions are distributed and interact can be important for implementing gene-drive technologies.

The factors determining the type of dynamics predicted by the model fall into three broad classes. First, physical environmental conditions are important, in particular the severity of the dry season which increases the probability of local extinction, leading to metapopulation dynamics. Previous models of driving-Y chromosome dynamics also found seasonality to be important and recommend that releases should be made early in the rainy season so as to capitalise on a period of rapid population growth [17, 18]. Second, ecological factors are important, in particular the rate of local dispersal. We find overall suppression always increases with dispersal rate, but with the dynamics leading to the coexistence of the two types of chromosome being most likely for intermediate dispersal rates. A similar result was found in a study of disease spread in metapopulations where the stable coexistence of uninfected and infected populations was most frequent at intermediate rates of host dispersal [40]. Very few studies have measured mosquito dispersal in West Africa and these suggest relatively high dispersal rates [37–39], but more data would be valuable. Finally, properties of the driving-Y chromosome itself are important: a high cleavage rate results in greater local population suppression but can also give rise to metapopulation dynamics so that overall suppression is highest at an intermediate cleavage rate, which is in agreement with previous spatial models [16, 17].

We chose to base our analysis on a programme of random release sites each year, in order to concentrate on the role of release intensity without being tied to a specific release scenario. Simulations where the release sites were arranged on an approximate lattice resulted in somewhat greater suppression than randomised releases. In an area of the size we simulate, concentrating resources in the locations with the most pronounced seasonality at the beginning of the rainy season and to less seasonal locations at other times of the year, would help maximise the spread of the driving-Y chromosome. Other possible improvements to a release programme could be investigated; for example, release sites might be selected on the basis of surveys to determine where wildtype mosquitoes are most abundant.

Unlike previous large-scale models of mosquito dynamics which assume a regular lattice of populations [41–43], we describe the ensemble population as a network of local populations associated with human settlements. While this approach ensures that the model captures the inherent heterogeneity of connectedness among mosquito populations, in comparison to lattice models it relies more heavily on the assumption that the target vector species is anthropophilic. The model is motivated by the biology of *An. gambiae* and its sister species *An. coluzzii*, which are both known to be strongly anthropophilic [20–23, 44], though the existence of populations outside of human settlements cannot be ruled out. There is a third important malaria vector in the *An. gambiae* complex, *An. arabiensis*, which is also present in the region [45]. This species varies in its degree of anthropophily [20], though is thought to be primarily associated with humans in the West African part of its range [46], and is found in drier habitats than *An. gambiae* or *An. coluzzii* [20]. There are further differences between the ecology of the three species [47–50], for example *An. coluzzii* is more able than either *An. gambiae* or *An. arabiensis* to exploit irrigated paddy fields as larval habitat [51]. Our current understanding of the comparative ecology of the three species is not developed enough to allow us to model them individually which is a challenge for the future. The data we use on the geographical locations of settlements probably do not include all human settlements in the region. Depending on their locations, the presence of additional mosquito populations associated with these settlements will reduce the effective isolation of the most remote populations and thus accelerate driving-Y chromosome spread into these populations.

In addition to driving-Y chromosomes autosomal gene-drive constructs that spread by homing (the conversion of heterozygotes into homozygotes) are also in development in malaria vectors, both for population suppression [52, 53] and population replacement [54]. Previous modelling results suggest a homing-based suppression gene-drive will have a similar impact on a population as an equivalent driving-Y chromosome [14, 16, 17], yet there may be differences in large heterogeneous environments that the current type of model could help identify. It is not yet clear which type of DEG will be ready first for regulatory consideration, but one attractive feature of driving-Y chromosomes is that they can be designed so that the evolution of resistance via target site mutations is likely to arise slowly if at all. This is done by choosing endonuclease target site sequences within multiply repeated rRNA genes on the *An. gambiae* X-chromosome [55]. Nevertheless, in a large population, the possibility that target-site resistance to a driving-Y chromosome might evolve cannot be discounted, and resistance may also evolve via mutations that create trans-acting autosomal suppressor alleles [56]. If a fully or relatively fit resistant genotype does establish in a connected part of a landscape, it is likely to eventually spread across the region. Previous modelling has highlighted a range of strategies to increase the time it will take for resistance to a driving-Y to establish, such as the simultaneous release of a second construct that is sufficiently different to the first that resistance to one does not confer resistance to the other [56], and this could be investigated further using the current modelling approach.

## Conclusions

The genetic modification of a mosquito vector is a novel approach to disease control and must be subject to rigourous and independent scrutiny to ensure it is safe for humans and for the environment. A crucial component of this process is understanding mosquito population dynamics after the release of a construct. In this study, we have investigated the potential of driving-Y chromosomes to suppress mosquito populations across an area of West Africa that is large enough to exhibit the wide variety of environmental conditions found in this region. Our results suggest that releases will result in population suppression in some regions and population elimination in others, giving rise to a high average population suppression across the study area. There is a temporal as well as a spatial element to the variability, as some locations may undergo sporadic episodes of population elimination and colonisation. We have explored several factors which influence the local and non-local dynamics, finding the magnitude of seasonality to be particularly important. We hope these results are a useful contribution to wider discussions on the use of gene-drive technology.

## Methods

### Overview

We extend a regional mosquito population model [19] by incorporating the genetics of a driving-Y chromosome. We first give an overview of the mosquito model here and refer the reader to ref. [19] for full details. The model uses settlement data collected by the United Nations Office for the Coordination of Human Affairs [57], inland water data extracted from the digital chart of the world [58], and rainfall data from the “ERA-interim reanalysis” (available from the European Centre for Medium-Range Weather Forecasts [59]). The default model parameters are given in Additional file 6: Table S1.

### The population network

The model assumes that mosquito populations are associated with humans, so that the overall population is a network of local populations at the sites of human settlement. At any time, each local population is described by the numbers of juveniles (both male and female), adult males, unmated adult females, and mated adult females. Each population is updated daily with different events—births, deaths, juvenile maturation, mating, dispersal—being determined by stochastic processes.

### Local population dynamics

Each female lays a Poisson-distributed number of eggs each day. All mosquitoes suffer mortality with a probability that we assume is independent of mosquito age, though differs between juveniles and adults. This mortality represents the effects of “background” risk factors such as predation and desiccation. In addition to background mortality, juveniles also suffer density-dependent mortality due to larval competition for resources. The severity of density-dependent mortality depends on the extent of breeding habitat in a location, which we assume is determined by the amount of rainfall that has fallen locally in the previous week, and the distribution of local water bodies. The juvenile stage is of fixed duration, after which the individual becomes an adult male or unmated female as determined by sex chromosome genetics. Unmated females become mated on a given day with a probability that depends on the number of adult males in the population. We choose parameters so that most females mate within 1 or 2 days unless the population is very small. Every adult male and mated female has the same probability of dispersing each day. On dispersing, an individual is assigned to a new site, which is selected from a local neighbourhood with probability that decreases with distance from the focal site.

### Aestivation and migration

To model aestivation, we assume mated adult females have a probability of entering a dormant state at the end of each rainy season (over a 50-day period). Many of these females will not survive aestivation, but those that do re-emerge to resume their activities at the beginning of the following rainy season (over a 30 day period). To model long-distance migration, we assume there is a period each year when mated adult females have a probability to migrate in a *SW*→*NE* direction and a second period when migration may occur in the opposite direction. A migrating female, if she survives, is redistributed to a new site chosen at random from the region that extends from the focal site to the edge of the simulation area in the given direction. The timing and direction of long-distance migration were chosen to reflect annual wind-patterns in this region [60].

### Driving-Y chromosomes

To incorporate driving-Y chromosomes, we classify all males by whether they carry a normal or driving Y-chromosome. We assume the normal Y is fixed in the global population prior to releases. When a female mates, the Y-chromosome of her mate is Bernoulli distributed with driving-Y probability equal to the frequency of driving-Y among all adult males in her local population (we assume that driving-Y males are as competitive in mating as normal-Y males). The mated female is then classified according to her mate’s Y-chromosome, which is inherited in all her male offspring (we assume that females only mate once). If a female is mated to a normal Y-chromosome male, each of her eggs is equally likely to be male or female. If her mate had a driving-Y chromosome, however, the expected number of male eggs per day is scaled up by the factor 1+*e* and female eggs down by the factor 1−*e* where *e* is the X-chromosome cleavage rate.

## Additional files

Additional file 1 **Figure S1**. A uniform release programme. The 424 release locations used to compare a stratified release programme against the default of random release sites. All releases are at the location of a settlement. (PDF 60 kb)

Additional file 2 **Figure S2**. Releases after 4 years are ineffective. The number of female mosquitoes through time, if releases are at the default intensity of 10 male mosquitoes at each of 424 sites per year (1% of sites). Colour bands show the 95% central quantile among simulation replicates. (PDF 10 kb)

Additional file 3 **Figure S3**. The effect of dispersal rate on the spatial distribution of genotypes. Left is after 4 years and right after 8 years. (JPG 1940 kb)

Additional file 4 **Figure S4** The effect of dry season ecology on the fraction of different population types. Bars show the fraction of each population type 4 years after driving-Y releases begin. (PDF 7 kb)

Additional file 5 **Figure S5** A spatially heterogeneous population growth rate does not affect the results. The figure uses a version of the model where the egg-laying rate (parameter *θ*, eggs per day per mated female) depends on groundwater and so varies in space and time, in the same way that larval competition does in our standard model. The assumption is that egg-laying rate can increase from a minimum *θ*_0_ to a maximum *θ*_0_+*θ*_1_+*θ*_2_ when there is a high rainfall and a high local density of water courses. This is in addition to the assumed affect of water on larval competition. Varying the baseline egg laying rate somewhat increases the predicted suppression (compare colours), yet allowing the egg-laying rate to increase in response to groundwater has no effect (x-axis). (PDF 10 kb)

Additional file 6 **Figure S6** Model parameters. (PDF 2 kb)
