# Supplementary figures and images for: Modelling the potential of genetic control of malaria mosquitoes at national scale
Source: BMC Biol. 2019 Mar 29;17:26. doi: 10.1186/s12915-019-0645-5 (PMC6440076; doi:10.1186/s12915-019-0645-5)

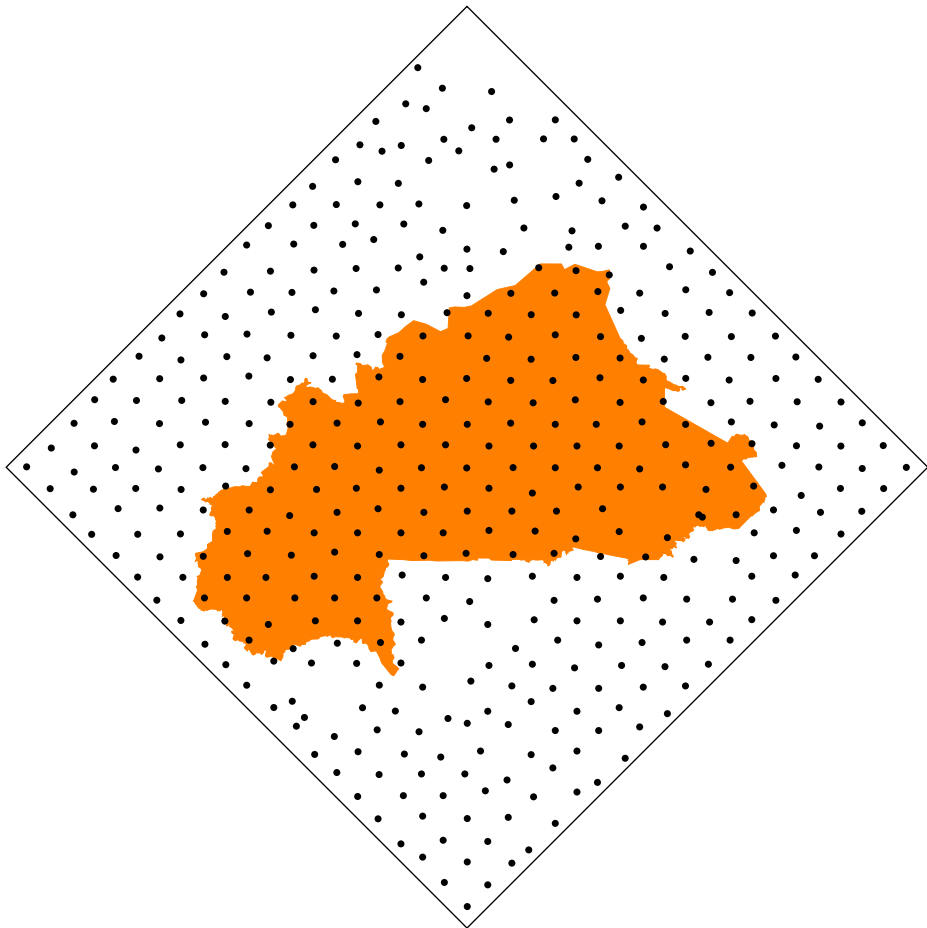

Supplement: Supplementary file 1 — Figure S1. A uniform release programme. The 424 release locations used to compare a stratified release programme against the default of random release sites. All releases are at the location of a settlement. (PDF 60 kb) [file 12915_2019_645_MOESM1_ESM.pdf]

■ No releases ■ Releases

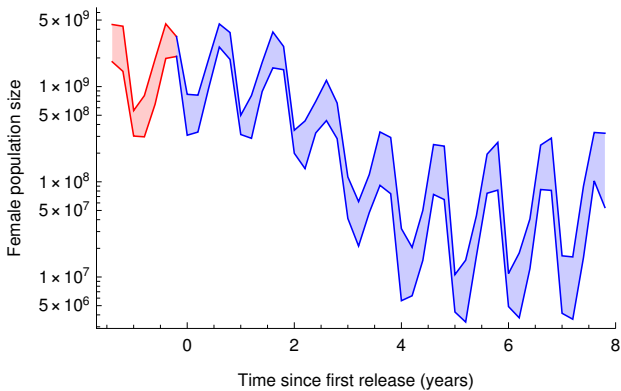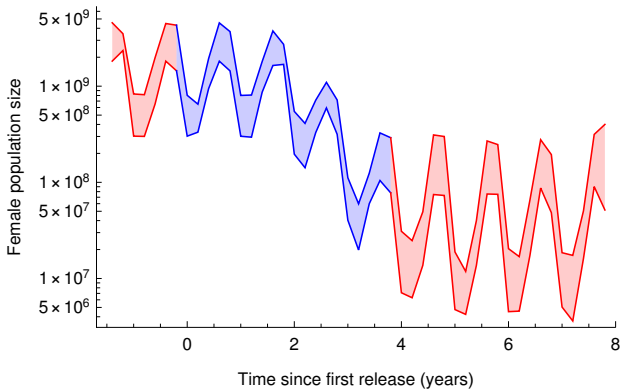

Supplement: Supplementary file 2 — Figure S2. Releases after 4 years are ineffective. The number of female mosquitoes through time, if releases are at the default intensity of 10 male mosquitoes at each of 424 sites per year (1% of sites). Colour bands show the 95% central quantile among simulation replicates. (PDF 10 kb) [file 12915_2019_645_MOESM2_ESM.pdf]

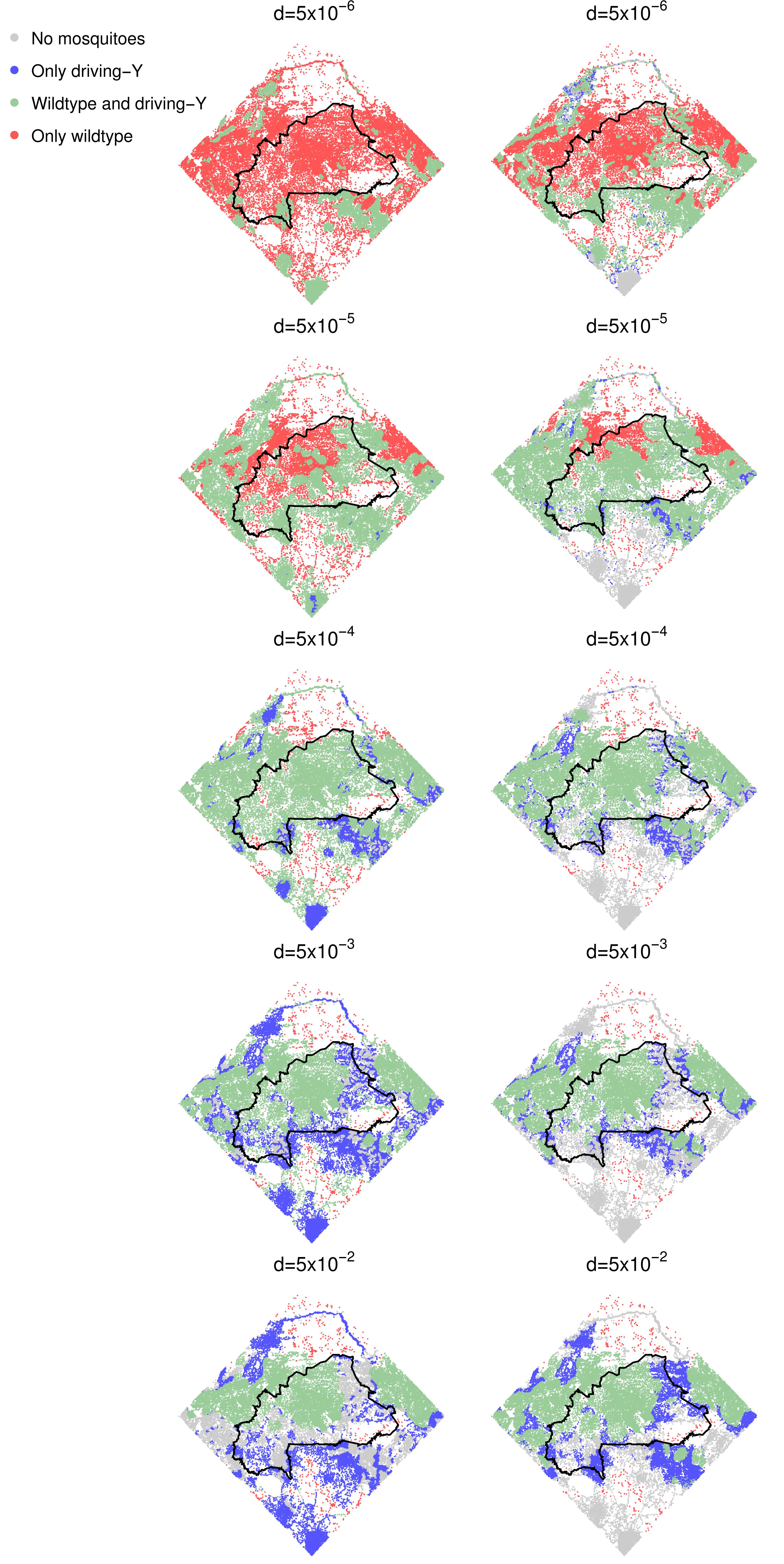

Supplement: Supplementary file 3 — Figure S3. The effect of dispersal rate on the spatial distribution of genotypes. Left is after 4 years and right after 8 years. (JPG 1940 kb) [file 12915_2019_645_MOESM3_ESM.jpg]

Proportion of sites

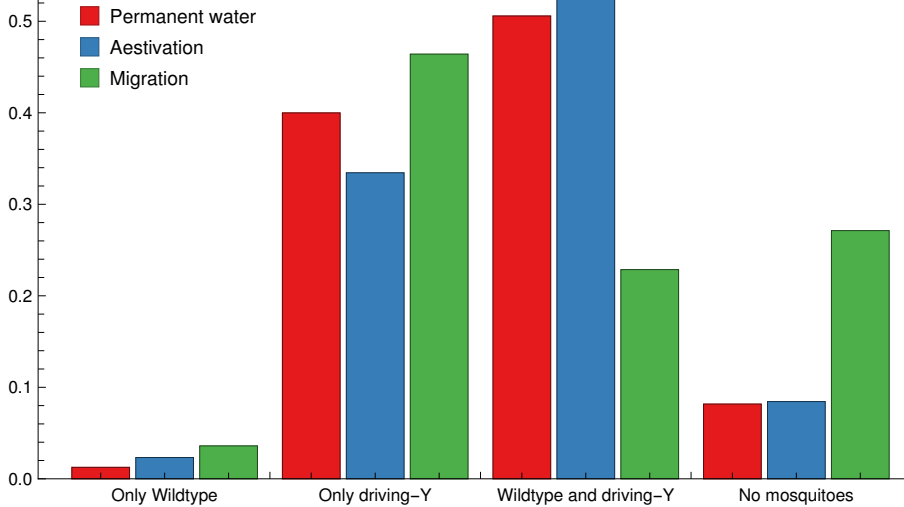

Supplement: Supplementary file 4 — Figure S4 The effect of dry season ecology on the fraction of different population types. Bars show the fraction of each population type 4 years after driving-Y releases begin. (PDF 7 kb) [file 12915_2019_645_MOESM4_ESM.pdf]

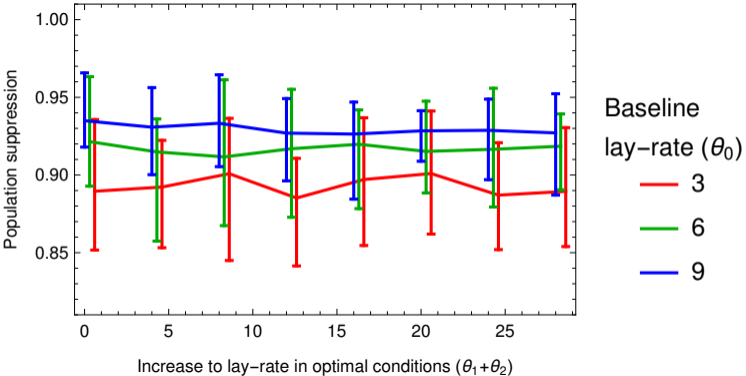

Supplement: Supplementary file 5 — Figure S5 A spatially heterogeneous population growth rate does not affect the results. The figure uses a version of the model where the egg-laying rate (parameter θ, eggs per day per mated female) depends on groundwater and so varies in space and time, in the same way that larval competition does in our standard model. The assumption is that egg-laying rate can increase from a minimum θ0 to a maximum θ0+θ1+θ2 when there is a high rainfall and a high local density of water courses. This is in addition to the assumed affect of water on larval competition. Varying the baseline egg laying rate somewhat increases the predicted suppression (compare colours), yet allowing the egg-laying rate to increase in response to groundwater has no effect (x-axis). (PDF 10 kb) [file 12915_2019_645_MOESM5_ESM.pdf]
